# Four new Neotropical species of *Eudicrana* Loew (Diptera, Mycetophilidae, Sciophilinae) from the Colombian high Andean ecosystems, with comments on the genus

**DOI:** 10.3897/zookeys.988.49627

**Published:** 2020-11-06

**Authors:** Carolina Henao-Sepúlveda, Marta Wolff, Dalton de Souza Amorim

**Affiliations:** 1 Grupo de Entomología, Universidad de Antioquia, Calle 67 # 53–108, Medellín, Colombia Universidad de Antioquia Medellin Colombia; 2 Departamento de Biologia, Faculdade de Filosofia, Ciências e Letras de Riberão Preto, Universidade de São Paulo, Avenida Bandeirantes 3900, 14040-901, São Paulo, Brazil Universidade de São Paulo São Paulo Brazil

**Keywords:** Andean ecosystem, biology, diversity, Neotropical region, taxonomy

## Abstract

Four new species of the sciophiline genus *Eudicrana* Loew are described for the Eastern and Central Andes of Colombia–*Eudicrana silvaandina***sp. nov.**, *E. chingaza***sp. nov.**, *E. maculata***sp. nov.** and *E. merizaldei*. These are the first species of *Eudicrana* described from the extreme northern range of the Andes. The altitudinal distribution of these species in the paramos and high Andean forest ecosystems is restricted to 1750–3660 m a.s.l. and some other information on the environment is briefly discussed. A key for the Colombian species of *Eudicrana* is provided and a discussion is elaborated on the position of these species within the genus.

## Introduction

The genus *Eudicrana* was originally proposed by Loew (1869) based on a single female of the type species of the *E. obumbrata*, from North America. The genus is also known from 11 other species: *E. nigriceps* Lundström from Europe (Lundström 1909) and *E. affinis* Okada from Japan (Okada 1938), *E. monticola* Tonnoir and *E. nicholsoni* Tonnoir from Australasian (Tonnoir 1929) and *E. araucariae* Matile, from New Caledonia (Matile 1991), and six Neotropical species–*E. basinerva* Freeman, *E. pallida* Freeman and *E. similis* Freeman, from the Argentinian Patagonia and southern Chile (Freeman 1951), *E. claripennis* Edwards and *E. vittata* Edwards, from Peru (Edwards 1931), and *E. splendens* Lane, from southeastern Brazil (Lane 1948). New combination– *Neuratelia elegans* (Lane, 1948)–was recently proposed for a Brazilian species *Eudicrana elegans* Lane by Henao-Sepúlveda et al. (2019).

*Eudicrana* clearly belongs to the Sciophilinae, distinguished from other genera by the lack of the mid ocellus, the lateral ocelli touching the eye margins, and the presence of R_4_ forming an elongated rectangular cell (Borkent and Wheeler 2013). In Borkent and Wheeler’s (2013) phylogenetic study of the Sciophilinae, *Eudicrana* appears as monophyletic in a clade also including *Polylepta* Winnertz and its sister genus, *Leptomorphus* Curtis. Both these genera share the presence of setae at the anterior part of the mediotergite. Analysis based on molecular data shows that *Eudicrana* is very close to *Sciophila*, but the studies are based on a very restricted taxon sampling of sciophiline genera (Ševčík et al. 2013; Kaspřák et al. 2018). Nearly nothing is known about its natural history.

**Figure 1.**
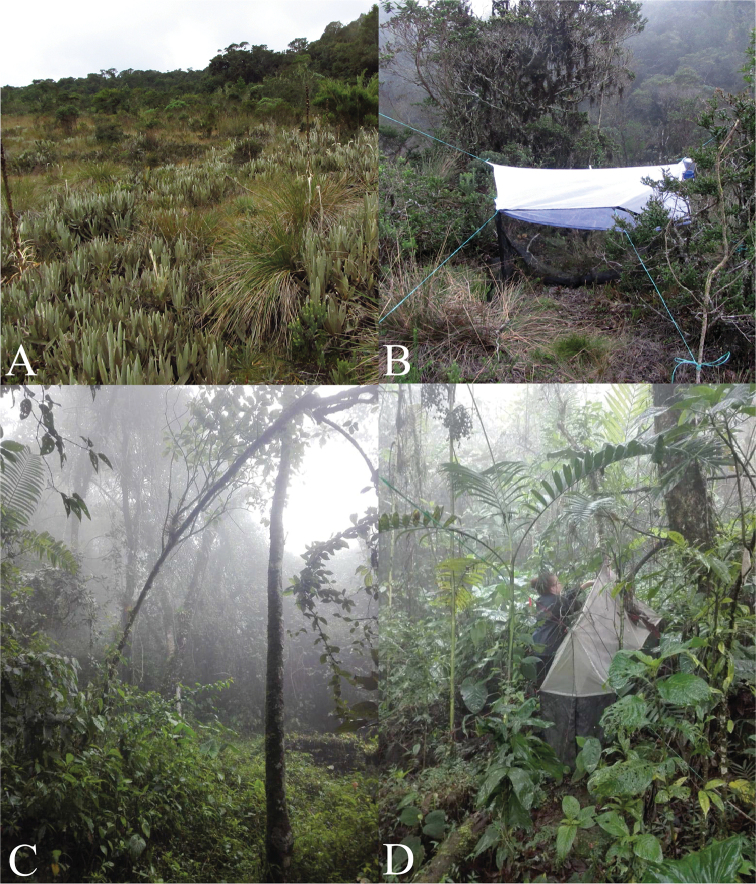
**A** Landscape images of the municipality of San José de la Montaña, paramo El Congo, Colombia, type locality of *Eudicrana maculata* sp. nov. (holotype) **B** Malaise trap habitat is in area covered on **A C** landscape images of the municipality of Envigado, private property Nubarrones, type locality of *E. merizaldei* sp. nov. (holotype) **D** Malaise trap habitat is in area covered on **C**.

The genus *Eudicrana* is one of the least studied genera of sciophilines. There has been no revision of the genus, despite the relatively low number of species, and the descriptions have very few illustrations. The Palearctic species, *E. obumbrata* has wing and terminalia illustrations (Lundström 1909: figs 155, 156; Johannsen 1910: figs 83,111; Fisher 1937: plate 11 fig. 1, Vockeroth 1981: fig.14.41). *E. nigriceps*, on the other hand, has only the terminalia illustrated by Hutson et al. (1980: fig. 188), who suggests that *E. obumbrata* and *E. nigriceps* may be synonymous due to slight differences between genitalia. In this case a slide montage or a new capture would be needed (Hutson 1979). Regarding the Australasian species, *E. nicholsoni* and *E. monticola*, the male terminalia are not illustrated; the last one has only the illustration of the wing by Tonnoir (1929: fig. 6), while Matile (1991: figs 7,8) illustrated the male terminalia and the wing of *E. araucariae*. Of the Neotropical species, *E. claripennis* and *E. vittata* were described based only on females and have no illustrations at all. Lane (1960: fig. 8) assigned a specimen from Trinidad to *E. vittata*. The holotype of *E. vittata* has been examined, as well as the specimen studied by Lane (1960), and they may not be conspecific. Freeman (1951) illustrated the male terminalia of the Chilean species in lateral view and included a photograph of the wing of *E. basinerva*, but important details of the terminalia are missing. Lane (1948) did not include an illustration of the wing of *E. splendens*.

This paper describes the first four known species of *Eudicrana* of the high Andean ecosystems of the Central and Eastern Cordilleras of Colombia. This includes illustrations of the head, thorax, wing and male terminalia for all four species, and the female terminalia for one of the species. The paper also intends to discuss the similarities between the species of the genus.

## Material and methods

The material studied here is deposited in the entomological collection Instituto de Investigación de Recursos Biológicos Alexander von Humboldt (IAvH-E) in Villa de Leyva, Boyacá, Colombia and the Colección Entomológica Universidad de Antioquia (CEUA), Medellin, Antioquia, Colombia.

The specimens were collected in pristine ecosystems using Malaise trap and sweeping net (Fig. 1A–D), preserved in 70% and 96% ethanol. One wing of the holotypes and/or paratypes was separated and mounted in permanent slide mountings with Euparal. The teminalia of the corresponding specimens were dissected and cleared in a solution of 10% KOH for 12 hours, then heated for 15 minutes, neutralized in acetic acid for 10 minutes, dehydrated in ethanol 70–96%, and preserved in a microvial in glycerine.

Photographs were taken using a Moticam 3.0 megapixel digital camera attached to an Olympus SZX7 stereomicroscope and with a Leica DFC500 camera coupled to a Leica M16 stereomicroscope. Photo stacking was performed using the Helicon Focus 6.7.2 software and edited with Adobe Photoshop CC 2017. Photographs and illustrations of the terminalia were prepared using the U–DA Olympus drawing tube attached to an Olympus BX40 compound microscope, then vectorized with Illustrator CC 2017.

Morphological terminology for head, thorax, pleural sclerites and terminalia follows Søli (1997), while terminology of the wing venation follows Amorim and Rindal (2007) and Cumming and Wood (2017).

### Abbreviations

**ae** = aedeagus; **ce** = cercus; **gc** = gonocoxite; **gc ap** = gonocoxal apodeme; **gc dl** = gonocoxite dorso-apical lobe; **gc dlp** = gonocoxite dorso-lateral projection; **gc vl** = gonocoxite ventral lobe; **gst** = gonostylus; **hyp** = hypandrium; **par** = paremeres; **par ap** = parameral apodeme.

## Results

### Genus Eudicrana

Taxon classificationAnimaliaDipteraMycetophilidae

Loew

558D0A97-3791-553C-93E6-F318FD826D99

Eudicrana Loew 1869: 142. Type species. Eudicrana obumbrata Leow (original designation).

#### Diagnosis.

(modified from Borkent and Wheeler 2013). Considerably large specimens, body elongate, mostly yellowish and/or brown. Two ocelli, in contact with eye margins. Laterotergite and mediotergite setose. Wing macrotrichia decumbent, some species without microtrichia. C clearly produced beyond apex of R_5_, sc-r reaching R_1_ almost always beyond origin of Rs, R_4_ present, forming an elongated rectangular cell, M_1+2_ short, barely twice length of r-m, M_1+2_ forking slightly more distally than origin of M_4_.

### Key to Colombian species of *Eudicrana* (males)

**Table:** 

1	Wing membrane with obvious dark maculae (Fig. 4C, D); anepisternum with a set of dorsal short hairs (Fig. 3G, H)	**2**
–	Wing membrane clear or with faint darkened areas along anterior margin but no obvious maculae (Fig. 4A, B); anepisternum bare (Fig. 3E, F)	**3**
2	Vein sc-r almost aligned with Rs (Fig. 4C); terminalia longer than wide and cercus almost twice the length of the gonocoxite.(Fig. 7D)	***E. maculata* sp. nov.**
–	Vein sc-r not aligned with Rs (Fig. 4D); terminalia wider than long and cercus once the length of the gonocoxite. (Fig. 8D)	***E. merizaldei* sp. nov.**
3	Terminalia with long, slightly inwards curved dorso-lateral projection of gonocoxite; small and rounded gonostylus; thin cerci (Fig. 5B)	***E. silvaandina* sp. nov.**
–	Terminalia with small dorso-lateral projection of gonocoxite; wide and lunular gonotylus; wide cerci (Fig. 6D)	***E. chingaza* sp. nov.**

### Eudicrana silvaandina sp. nov.

Taxon classificationAnimaliaDipteraMycetophilidae

6220EB67-7C58-5056-8F0B-DA6046813682

http://zoobank.org/FFB534C3-EDC9-44F2-8E63-07326DBD2D8D

**Figure 2.**
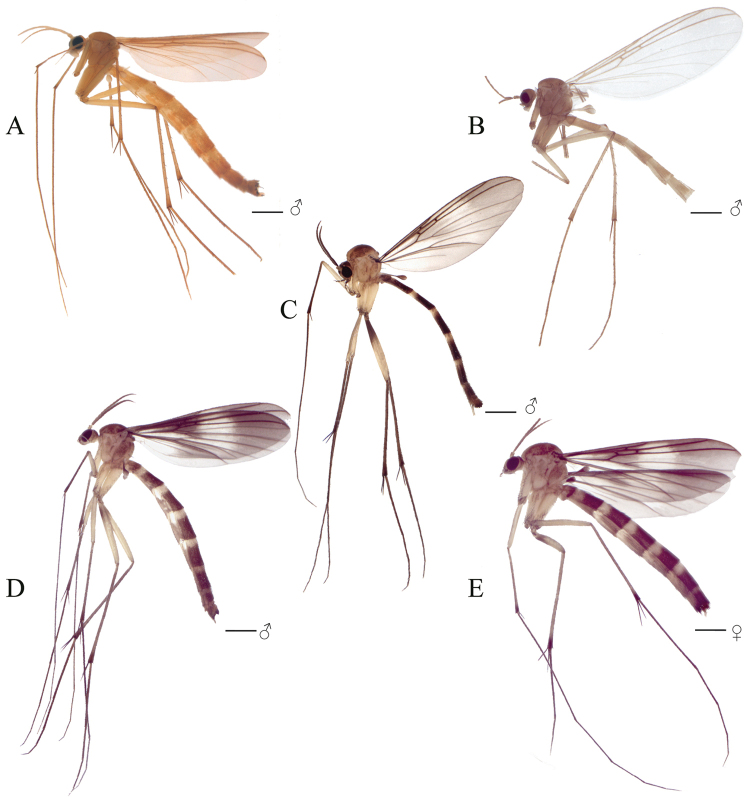
**A** Habitus of *Eudicrana silvaandina* sp. nov., male (holotype) **B** Habitus of *E. chingaza* sp. nov., male (holotype), terminalia detached **C** Habitus of *E. maculata* sp. nov., male (holotype) **D** Male habitus of *E. merizaldei* sp. nov. (holotype) **E** Habitus of *E. merizaldei* sp. nov., female. Scale bar: 1mm

**Figure 3.**
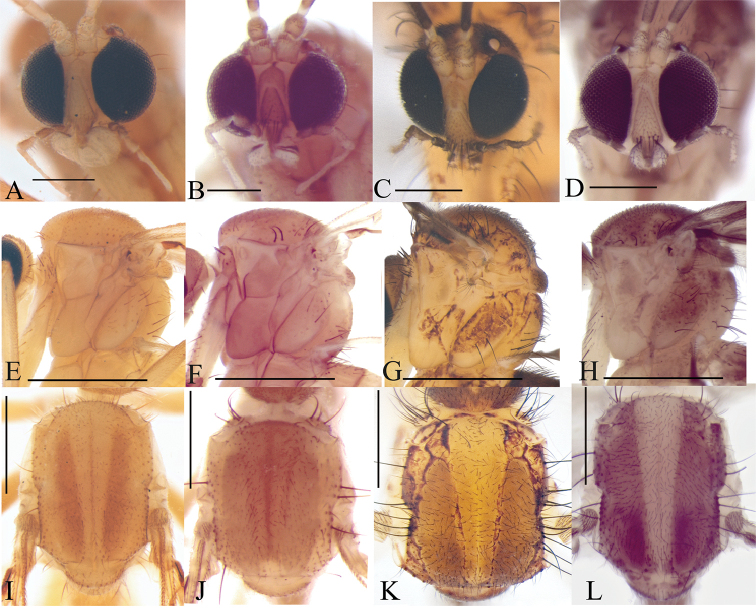
Head, frontal view **A***Eudicrana silvaandina* sp. nov. (holotype) **B***E. chingaza* sp. nov. (holotype) **C***E. maculata* sp. nov., (holotype) **D***E. merizaldei* sp. nov. (holotype). Thorax, lateral view **E***Eudicrana silvaandina* sp. nov. (holotype) **F***E. chingaza* sp. nov. (holotype) **G***E. maculata* sp. nov. (holotype) **H***E. merizaldei* sp. nov. (holotype). Thorax, dorsal view **I***Eudicrana silvaandina* sp. nov. (holotype) **J***E. chingaza* sp. nov. (holotype) **K***E. maculata* sp. nov. (holotype) **L***E. merizaldei* sp. nov. (holotype). Scale bar: 0.25 mm.

**Figure 4.**
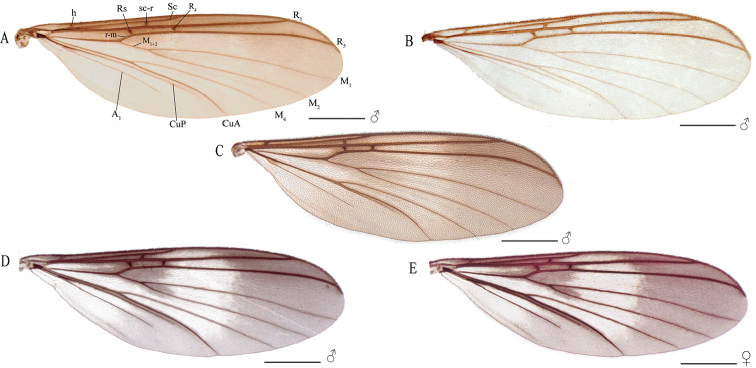
**A** Wing of *Eudicrana silvaandina* sp. nov. (holotype) **B** wing of *E. chingaza* sp. nov. (holotype) **C** wing of *E. maculata* sp. nov. (holotype) **D, E** wing of *E. merizaldei* sp. nov. **D** male (holotype) **E** female (paratype). Scale bar:1 mm.

**Figure 5.**
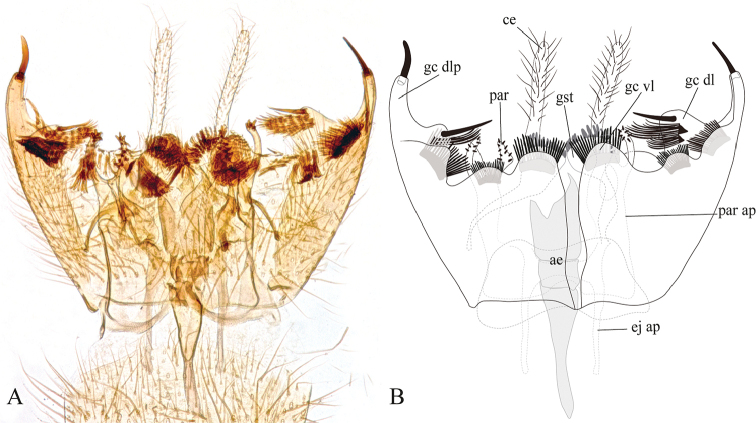
Male terminalia of *Eudicrana silvaandina* sp. nov. (holotype), ventral view **A** photograph **B** drawing.

Figs 2A , 3A, E, I , 4A , 5

#### Type material.

***Holotype*.** 1♂, Colombia, Department of Cundinamarca, Chingaza National Natural Park (PNN), Alto de la Bandera locality; 4°34.351'N, 73°42.752'W; alt. 3660 m a.s.l.; forest; Malaise trap; L. Cifuentes leg.; (IAvH 2600, wing in Euparal on slide mounting, rest of the body in 96% ethanol, genitalia preserved in glycerine in microvial). ***Paratype*.** 1♂, Colombia, same data as holotype. (CEUA 11339, in alcohol).

#### Diagnosis.

General color yellow to light brown. Anepisternum bare. All coxae and hind femur with no dark markings. Wing darker along anterior margin, but without conspicuous maculae; sc-r beyond origin of Rs. Terminalia yellowish, wider than long. Lateral extension of gonocoxite long, slightly curved inwards, with an apical long dark spine. Gonostylus small and rounded, apical surface with scattered short spines. Parameres digitiform, apically bifurcated, with short dark spines.

#### Description.

***Male*** (Fig. 2A). Body length, 8.0–8.5 mm. *Head* (Fig. 3A). Width, 0.56 mm, height, 0.35 mm. Vertex and occiput yellowish, darkened around the ocelli, with abundant brownish-yellow short setae. Mid ocellus absent, lateral ocelli nearly touching eye margins. Eyes setose. Four long dark setae on occiput behind eye margin. Scape and pedicel yellow, cylindrical, scape slightly longer than pedicel, both with small brownish-yellow setae; 14 flagellomeres, mostly light brown; first flagellomere almost twice as long as second. Frons yellowish, setose; face yellowish, circular and setose; clypeus yellowish-brown, slightly elongate, sub-triangular, with abundant yellowish setae; palpus with palpifer plus four palpomeres, first parlpomere as long as second, light brown, distal flagellomeres gradually lighter, distal palpomere more than three times as long as penultimate. Labella well developed, cream-yellow. *Thorax* (Figs 3E, I). Scutum mostly yellowish, with a pair of elongated brownish stripes and a brownish line over acrostichals. Dorso-centrals slightly stronger than scattered setae over scutum, acrostichals undifferentiated from scutum setae, a number of stronger and longer black setae along lateral margins. Scutellum, yellowish, with scattered smaller setae over disc and two pairs of marginal setae slightly longer than remaining scutellar setae. Pleural sclerites yellowish brown, membrane pale-yellow. Antepronotum with four long darker setae, proespisternum with two stronger setae and some smaller ones. Proepimeron, anepisternum, katepisternum, mesepimeron, and metepisternum bare, laterotergite with 7–8 short setae on anterior half and 9–10 longer, darker setae on posterior half; mediotergite with longer dark setae along entire surface, dorsomedial setae shorter. Halter pedicel and knob yellowish, setose. *Legs*. Very elongate, yellowish brown, darker toward tip of femora and tibiae. Fore tibia with distal ventral oval depression with abundant and irregularly distributed trichia; first tarsomere 1.5 times tibia length. Mid tibia with short dark trichia irregularly arranged, with an apical ventral dark comb of setae, tarsi with dark, short, erect setae along entire length. Hind tibia with trichia as in mid tibia, but apical comb absent. Tibial spurs 1:2:2, light brown, spurs more than three times apical width of tibiae. Tarsal claws with a large apical tooth and a smaller basal tooth. *Wing* (Fig. 4A). Length 5.0–5.5 mm, width 2.0 mm. Membrane mostly hyaline, no defined maculae, but darkened along anterior margin, densely covered with decumbent macrotrichia on all cells and scarce microtrichia on anal lobe; wing veins light brown, anterior veins more strongly sclerotized. Sc complete, setose, reaching C slightly beyond level of R_4_; sc-r present, bare, slightly more basal than mid of cell r1; first sector of Rs slightly oblique, R_1_ long reaching C at about apical fifth of wing; C extending to slightly beyond tip of R_5_, R_4_ present, cell r1 rectangular, elongate, 8 times as long as wide; R_5_ slightly curved posteriorly at apex; r-m setose, oblique. Medial and cubital veins complete, basally reaching wing margin, though not hardly sclerotized distally. M_1+2_ stem almost twice the r-m length, M_1_ slightly divergent from M_2_ close to apex. Origin of M_4_ more basal than level of medial fork; CuA curved towards posterior margin on apical third; pseudovein sclerotized to about third of CuA; CuP sclerotized to about mid of CuA. *Abdomen*. (Fig. 2A). Cylindrical with dark setae covering tergites and sternites; segments 1–6 yellowish brown, darker on distal half, 7–8 brownish. *Terminalia* (Fig. 5A, B). Light brown, wider than long. Gonocoxites almost fusing to each other mesally at ventral face, with a deep, slender incision between them; each gonocoxite with three rounded ventral lobes over the distal margin, inner surface covered with a set of homogeneous combs of dark setae. Dorsal lobe of gonocoxite, slightly extended inwards, inner surface covered with a set of combs of elongated setae and a long strong dark subapical spine; dorso-lateral projection of gonocoxite long, extending to about half the cercus length, slightly curved inwards, bearing a long dark spine at tip. Gonocoxal bridge strongly displaced towards base of terminalia. Gonostylus, apically round, surface with scattered dark short spines. Paramere wide, bifid with a pair of digitiform distal projections with short dark spines. Aedeagus elongate, weakly sclerotized. Cerci digitiform, very long, slender and setose.

***Female*.** Unknown.

#### Etymology.

The specific epithet of this species combines the Latin word *silva* (nominative, noun, feminine) for “forest”, with *andina* (nominative, adjective feminine) for “Andean”, referring the presence of this species in the South American Andean ecosystem.

#### Remarks.

This species is easily discriminated from the other species described here by the faint darkened wing membrane along the entire anterior margin, the elongated, thin cerci, the long dorso-lateral projection of gonocoxite extending well beyond the gonostyle, bearing a distal spine, and the parameres with a pair of distal spinose projections.

### Eudicrana chingaza sp. nov.

Taxon classificationAnimaliaDipteraMycetophilidae

8BD96A49-2A74-5B36-A23A-AAA0CC3DE523

http://zoobank.org/8B41EC8-204B-4655-9B11-FE5069403094

**Figure 6.**
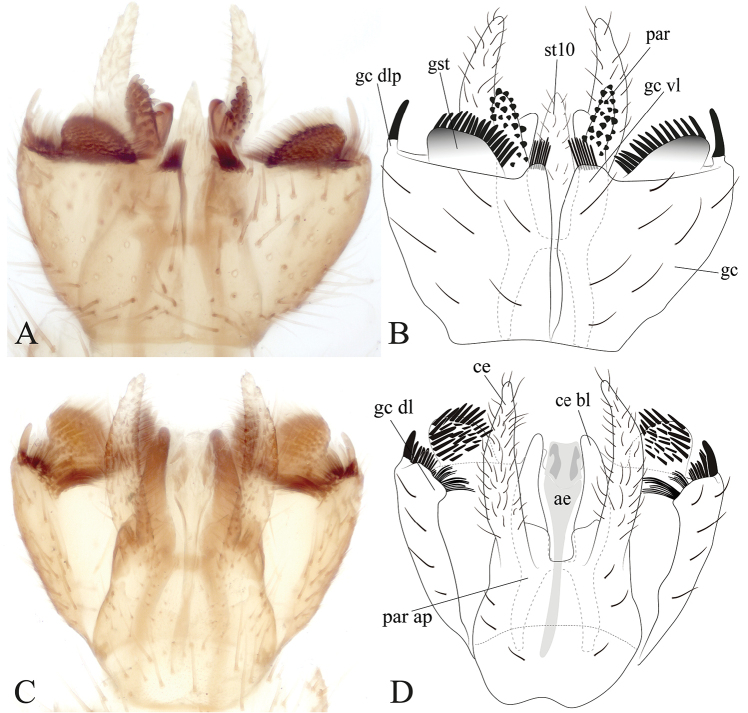
Male terminalia of *Eudicrana chingaza* sp. nov. (holotype) **A, B** ventral view **C, D** dorsal view **A, C** photograph **B, D** drawing.

Figs 2B , 3B, F, J , 4B , 6

#### Type material.

***Holotype*.** 1♂, Colombia, Department of Cundinamarca, Chingaza National Natural Park (PNN), Alto de la Bandera locality; 04°34.351'N, 73°42.752'W; alt. 3660 m a.l.s.; 15 Nov.–01 Dec. 2001; forest; Malaise trap; L. Cifuentes leg. (IAvH 2600, wing in Euparal on slide mounting, rest of the body in 96% ethanol, genitalia preserved in glycerine microvial).

#### Diagnosis.

Body light brown. Anepisternum bare. Coxae and hind femur without maculae. Wing membrane translucent; sc-r beyond of origin of Rs. Terminalia yellowish, wider than long. Dorso lateral-distal extension of gonocoxite short, with a distinctive apical spine. Gonostylus wide and lunular, inner surface with abundant spines. Cercus elongate, but wide on basal half. Paramere not bifid, elongate, with short spines.

#### Description.

***Male*** (Fig. 2B). Body length, 7.0 mm. *Head* (Fig. 3B). Width, 0.60 mm, height, 0.37 mm. Vertex and occiput light brown, with abundant brownish-yellow short setae. Mid ocellus absent, lateral ocelli surrounded by dark brown, almost touching eye margin. Eyes setose. Four long dark setae on occiput behind eye margin. Scape and pedicel yellowish brown, cylindrical, scape slightly longer than pedicel, both with small brownish setae; 14 flagellomeres, first flagellomere almost twice as long as second. Frons yellowish brown, setose, face yellowish brown, elongate, setose; clypeus yellow, quite elongate, sub-triangular, with abundant brownish setae; palpus with palpifer plus four palpomeres, light brown, first palpomere as long as second, last palpomere more than three times as long as penultimate. *Thorax* (Fig. 3F, J). Scutum light brown, with a narrow brown stripe along acrostichal line and a pair of weak light brown slender band over dorsocentral lines; dorsocentrals present, slightly longer than other scattered setae on scutum, acrostichals not differentiated, a number of stronger and longer black setae along lateral margins. Scutellum yellowish brown, with scattered setae along distal margin. Pleural sclerites yellowish brown, ventral half of katepisternum slightly darker. Pleural membrane pale-yellow. Antepronotum with four strong, darker setae; proespisternum with a pair of darker setae. Proepimeron, anepisternum, katepisternum, mesepimeron, and metepisternum bare; laterotergite with shorter setae on anterior half and 9–10 long setae on posterior half; mediotergite with lateral longer dark setae along entire surface and dorsomedial setae shorter. Halter pedicel yellow, knob light brown, setose. *Legs*. Coxae brownish-yellow, femora, tibia, and tarsi light brown, darkened towards apex [femur, tibia and tarsus of front leg missing in the holotype]. Mid tibia with short setation irregularly arranged, with a distal comb ventrally and some dark, slightly longer setae laterally and ventrally [tarsi missing]; hind tibia trichia distributed as on mid tibia, with dark slightly longer setae laterally and dorsally, without apical ventral comb. Tibial spurs 1:2:2, light brown, hind spurs more than three times apical tibial width. Tarsal claws with large apical tooth, smaller basal tooth. *Wing* (Fig. 4B). Length 5.0 mm, width 2.0 mm. Membrane very light brown, no maculae, densely covered with decumbent macrotrichia on nearly all wing cells, and scarce microtrichia on anal lobe; veins brown. Sc complete, setose, reaching C well slightly beyond level of R_4_; sc-r present, bare, just basal to mid of cell r1; first sector of Rs slightly oblique, R_1_ long, reaching C at apical fifth of wing, C extending slightly beyond apex of R_5_, R_4_ present; cell r1 elongate, rectangular, setose, length about 8 times the width; R_5_ gently curved at apex towards posterior margin. Medial and cubital veins complete basally, slightly less sclerotized close to margin. M_1+2_ about twice r-m length, M_1_ almost parallel to M_2_ distally. Origin of M_4_ more basal than level of medial fork. CuA curved towards wing margin at distal third; pseudovein sclerotized to distal third of CuA; CuP sclerotized to about mid of CuA. *Abdomen* (Fig. 2C) Segments light yellowish brown, distal two thirds darker, cylindrical, brownish setae covering tergites and sternites. *Terminalia* (Fig. 6A–D). Light brown, wider than longer. Gonocoxite almost fusing basally at ventral face, with a deep, slender incision between them; each gonocoxite with one distal ventral lobe at the distal margin, bearing a distal comb of straight dark setae. Dorsal lobe of each gonocoxite, slightly truncated at the apex, bearing at the inner surface scattered short spines and several set of combs of dark setae; dorso-lateral projection of gonocoxite short with a dark distal dark spine. Gonocoxal apodeme at about mid of terminalia. Gonostylus rounded and wider (lunular shape), inner surface bearing several and scattered short spines. Paramere digitiform, apical surface covered with short dark spines. Aedeagus elongate, weakly sclerotized and visible mesally. Cerci setose, digitiform and wider at basal half.

***Female*.** Unknown.

#### Etymology.

The specific epithet of this species *E. chingaza* (Nominative, adjective feminine) refers to Natural National Park Chingaza, where the holotype was collected. This name comes from the muisca indigenous language of Colombia, meaning “mountain range of the gods of the night”. It is one of the largest paramo ecosystems of Colombia and is the type locality of *E. chingaza*.

#### Remarks.

This species can be clearly separated from *E. silvaandina* by the short latero-distal projection of the gonocoxite in *E. chingaza*, by the wider cercus, and the nearly translucent wing membrane, without a darkened anterior margin of the wing. *E. maculata* also has a short latero-distal projection of the gonocoxite, but has a clear maculation in the wing and sc-r is placed very close to the origin of Rs.

### Eudicrana maculata sp. nov.

Taxon classificationAnimaliaDipteraMycetophilidae

02A10334-186E-5867-9819-18F4DE2957FD

http://zoobank.org/8BA9D52C-6931-41FC-9933-0531CC1ECCEB

**Figure 7.**
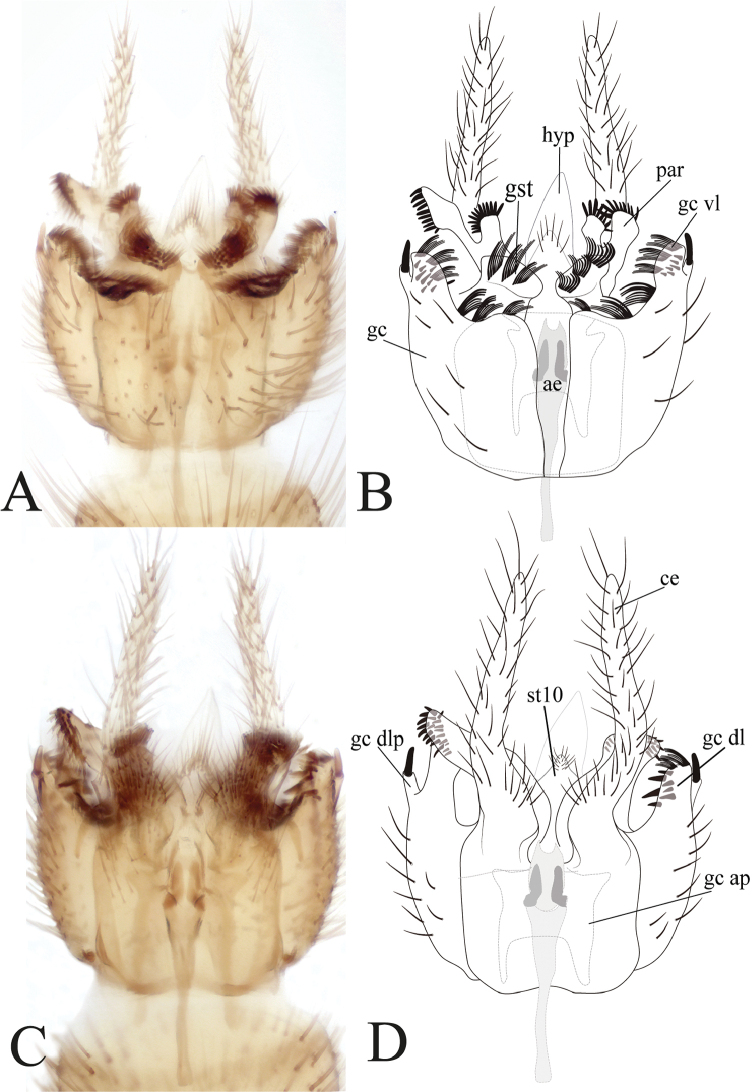
Male terminalia of *Eudicrana maculata* sp. nov. (holotype) **A, B** ventral view **C, D** dorsal view **A, C** photograph **B, D** drawing.

Figs 2C , 3C, G, K , 4C , 7

#### Type material.

***Holotype*.** 1♂, Colombia, Department of Antioquia, San José de la Montaña municipality, El Congo rural settlement, paramo El Congo locality; 6°46.5652'N, 75°43.5702'W, alt. 3150 m a.s.l.; 25 Mar.–5 Apr. 2017; Proyecto Moscas de las flores. C .H–Sepúlveda and M. Wolff legs.; dwarf forests, sweeping net (CEUA 106590, wing in Euparal on slide mounting, rest of the body in 96% ethanol, genitalia preserved in glycerine microvial). ***Paratypes*.** 1♂, same data as holotype (CEUA 106591, in alcohol); 1♂, Colombia Department of Antioquia, Sonsón municipality, paramo of Sonsón locality; 5°4.1292'N, 75°14.8092'W; alt. 3000 m a.s.l.; 10–15 Sept. 2011; forest; Malaise trap; L. Rios leg.; (CEUA 113393, in alcohol).

#### Diagnosis.

Scutum with a wide dark brown band. Anepisternum with a set of short dorsal setae. Coxae darkened distally, hind femur with a blackish brown marking on basal third. Wing with a dark brown macula along margin at region of sc-r, base of Rs and R_4_, r-m, bM, M_1+2_ and base of medial fork and over entire apical third. Abdomen blackish brown except in the basal third of each segment. Terminalia as long as wide, with short dorso-lateral projection of gonocoxite bearing a strong distal spine. Cerci very long, wide basally and setose. Paramere bifid, apically rounded and wide, with long dark spines on the margin.

#### Description.

***Male*** (Fig. 2C). Body length, 7.5–8.0mm. *Head* (Fig. 3C). Width, 0.55–0.57 mm, height, 0.33–0.35 mm. Vertex brown, with abundant short brownish setae. Mid ocellus absent, lateral ocelli almost touching eye margin and surrounding by dark brown. Eyes setose. Occiput with three long dark setae behind eyes. Scape slightly longer than pedicel, ochre-yellow cylindrical, both with abundant dark setulae on distal half; 14 flagellomeres dark brown, first flagellomere with basal fourth whitish yellow, almost twice as long as second. Frons light brown, setose; face yellowish brown, semi-circular, setose; clypeus yellowish brown, elongate, with shorter setae on dorsal half and a set of dark longer setae on ventral half; palpus with palpifer plus four palpomeres, brown, first palpomere about as long as second, last palpomere light brown, more than three times as long as penultimate. *Thorax* (Fig. 3G, K). Scutum with yellowish area mesally, delimited by weak marks at anterior and lateral margins, with a pair of light brown lateral bands, blackish brown markings spread over scutum; scutum covered with scattered small setae, dorsocentrals differentiated, slightly longer than remaining setae, acrostichals not differentiated, a number of stronger and longer black setae along lateral margins. Scutellum yellowish-brown on disc, with brown distal margin, scattered homogeneous short setae on distal margin, with some longer setae. Pleural sclerites mostly ochre yellowish, with dark brown irregular spots. Pleural membrane pale yellow. Antepronotum with six longer setae, proespisternum with shorter irregularly dark setae. Anepisternum with a dorsal set of short setulae. Proepimeron, katepisternum, mesepimeron, and metepisternum bare, laterotergite with shorter setae on anterior half and 10–12 long setae on posterior half; mediotergite with long lateral setae and a number of smaller dorso-medial setae. Halter pedicel and knob light brown, setose. *Legs*. Fore coxae and mid femora cream-yellow, coxae darker at tips; all tibiae and tarsi dark brown, hind femur blackish-brown on basal third. Fore tibia with distal oval depression ventrally with abundant, irregularly distributed trichia, first tarsomere 1.5 times tibia length. Mid tibia with short irregularly arranged setation and some dark setae, a ventral comb apically, tarsi with dark, short erect setae along entire length. Hind tibia with blackish short setae laterally and dorsally along tibia length, ventral comb absent. Tibial spurs 1:2:2, dark brown, spurs more than three times tibia width at apex. Tarsal claws with large apical tooth, smaller basal tooth. *Wing* (Fig. 4C). Length 5.5–6.0 mm, width 2.0 mm. Membrane light brown, densely covered with decumbent macrotrichia, microtrichia present; a slightly darker macula along margin at region of sc-r, base of Rs and R_4_, r-m, bM, M_1+2_ and base of medial fork and over entire apical third. Sc complete, setose, reaching C well beyond base of Rs, near to level of R_4_; sc-r present, bare, just slightly beyond level of origin of Rs; first sector of Rs only slightly oblique, setose; R_1_ long, reaching C at apical fifth of wing; C extending shortly over apex of R_5_; R_4_ present, cell r4 elongate, setose, about 5.7 times as long as wide; R_5_ slightly curved towards posterior margin at apex; r-m setose, oblique, shorter than M_1+2_. Medial and cubital veins complete basally. M_1+2_ twice length of r–m; origin of M_4_ more basal than medial fork; CuA gently curved towards base at apical third; pseudovein sclerotized to distal third of CuA; CuP sclerotized to about basal third of CuA. *Abdomen* (Fig. 2B). Abdominal segments 2-6 elongate, cream yellow on basal fifth, blackish brown on distal four fifth, dark setae covering tergites and sternites. *Terminalia* (Fig. 7A–D). Dark brown, wider than long. Gonocoxites almost fusing at ventral face, with a thin incision between them; dorsal margin covered with several combs of elongated setae; inner surface of the ventral lobe of gonocoxite bearing scattered short spines and several combs of setae. Dorsally without a pronounced lobe, over the inner surface covered with short and scattered spines; dorsal latero-distal extensions short, with a strong external subdistal spine. Gonostylus short and rounded, apical surface with a set of dark combs. Paramere with two distal projection bearing dark spines distally. Aedeagus triangular distally. Cercus digitiform, long, extending well beyond distal margins of gonocoxite, slender, slightly wider at base, setose.

***Female*.** Unknown.

#### Etymology.

The specific epithet *maculata* (nominative, adjective feminine) of this species refers to the evident dark brown maculae on the wing membrane.

#### Remarks.

This species is present in the transitional “dwarf forests” (Fig. 1B), between the high Andean forest and the paramos. This kind of environment is mainly dominated by the Melastomataceae species *Tibuchina grossa* (L.f) Cogn., the Cunoniaceae*Wienmannia tomentosa* L.f., the Clusiaceae*Clusia multiflora* Kunth, and the Winteraceae*Drimys granadensis* L.f.. This habitat is also rich in bryophyte mosses and the soil has a large layer of leaf litter. It is a very humid environment, usually with low temperature and low luminosity due to clouds.

### Eudicrana merizaldei sp. nov.

Taxon classificationAnimaliaDipteraMycetophilidae

1729B152-8CD1-5D4B-91F8-B64D45793897

http://zoobank.org/F27D7CCB-3843-4B7A-9C9A-301A73BA937B

**Figure 8.**
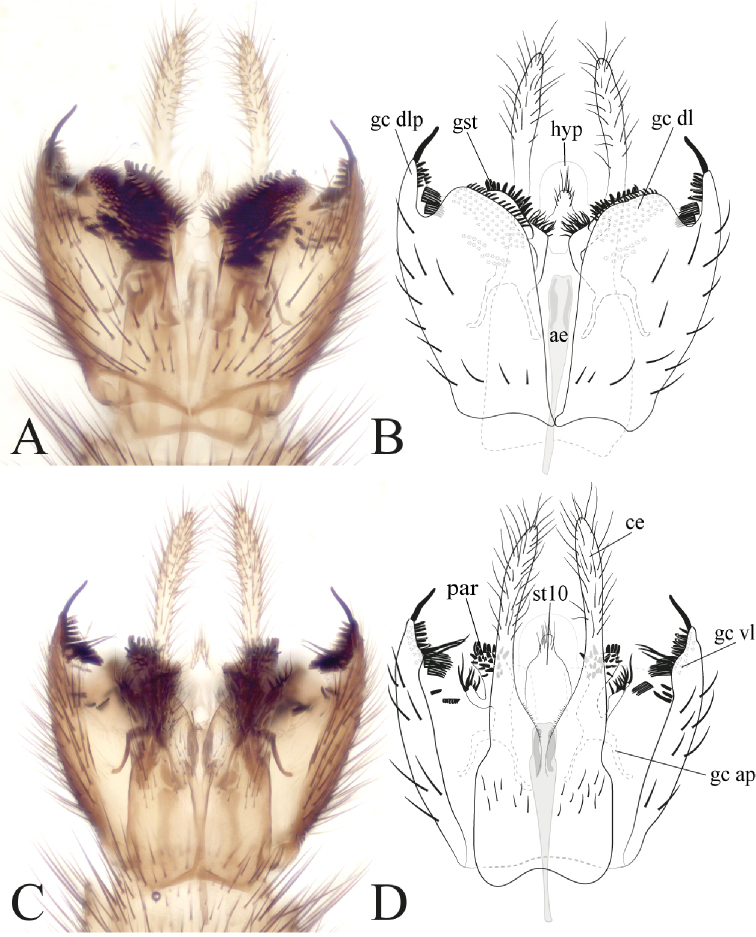
Male terminalia of *Eudicrana merizaldei* sp. nov. (holotype) **A, B** ventral view **C, D** dorsal view **A, C** photograph **B, D** drawing.

**Figure 9.**
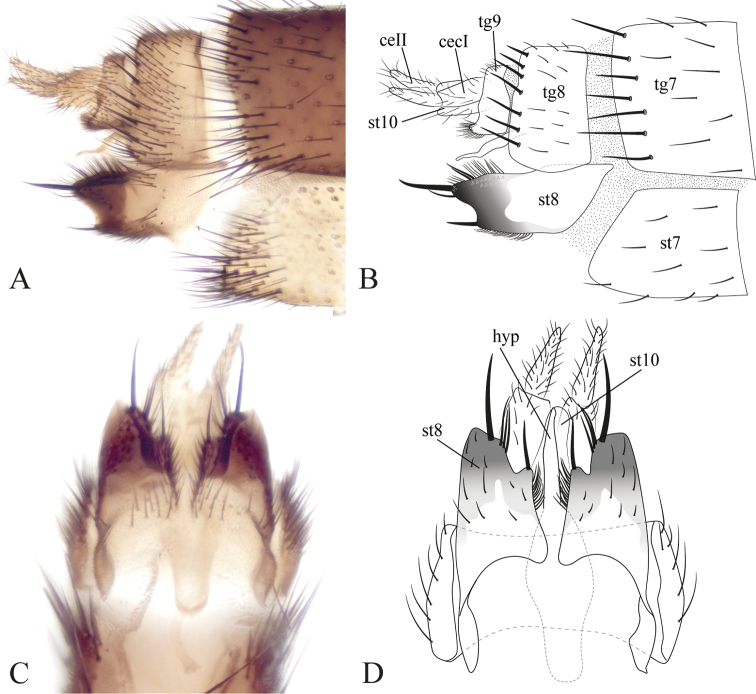
Female terminalia of *Eudicrana merizaldei* sp. nov. (paratype) **A, B** lateral view **C, D** ventral view **A, C** photograph **B, D** drawing.

Figs 2D , 3D, H, L , 4D , 8 , 9

#### Type material.

***Holotype*.** 1♂, Colombia, Department of Antioquia, Envigado municipality, private property Nubarrones; 6°8'45.626"N, 75°33'22.53"W; alt. 2200 m a.s.l.; 29 Jan.–11 Feb. 2017; M. Wolff and C. H-Sepúlveda; forest; Malaise trap (CEUA 108470, wing mounted in Euparal on microscope slide, rest of body in alcohol 96% ethanol). ***Paratypes*.** 2♂, 2♀, same location data as holotype, but differs on 26 Feb.–12 Mar. 2017 (CEUA 107038, in alcohol); 1♂, same location data as holotype, but differs on 23 Jun.–2 Jul. 2018 (CEUA 107040, in alcohol).

#### Diagnosis.

Body yellow to light brown. Anepisternum with a set of dorsal short setulae. Mid coxae and hind femur with dark markings. Wing with conspicuous maculae; sc-r reaching C beyond M_1+2_ fork, at apical third of wing. Terminalia yellowish, with dorsal margin dark brown, lateral rounded apical extension with apical long dark spine.

#### Description.

***Male*** (Fig. 2D). Body length, 7.5–8.5 mm. *Head* (Fig. 3D). Width, 0.54–0.56 mm, height, 0.30–0.35 mm. Vertex and occiput ochre-yellowish, light brown around ocelli, with abundant brownish-yellow short setae. Mid ocellus absent, lateral ocelli nearly touching eye margin. Eyes setose. Four long dark setae on occiput behind eye. Scape and pedicel ochre-yellowish, cylindrical, scape slightly longer than pedicel, both with small brownish setae; 14 flagellomeres, mostly light brown; first flagellomere almost twice as long as second. Frons and face ochre-yellowish, setose; clypeus yellowish-brown, slightly elongate, sub-triangular, with abundant light brown setae and some few darker setae; palpus with palpifer plus four palpomeres, first parlpomere as long as second, light brown, distal flagellomeres gradually lighter, distal palpomere more than three times as long as penultimate. Labella well-developed, cream-yellow. *Thorax* (Fig. 3H, L). Scutum with yellowish area between dorsocentrals, lateral margins, with a pair of light brown lateral bands; acrostichals indistinguishable from other setae, a number of stronger dark setae along lateral margins. Scutellum ochre-yellowish with light brown band along distal margin, scattered small setae over disc and two pairs of slightly longer marginal setae. Pleural sclerites ochre-yellowish, membrane cream-yellow. Antepronotum with three long dark setae, proespisternum with one stronger seta and some smaller ones. Anepisternum with a dorsal set of short setae, proepimeron, katepisternum, mesepimeron, and metepisternum bare, laterotergite with short 9–10 shorter setae on anterior half and 9–12 longer, darker setae on posterior half; mediotergite with longer dark setae along entire height, dorsomedial setae shorter. Halter pedicel light brown, knob yellowish, setose. *Legs* (Fig. 2D–E). Legs very elongate, ochre-yellowish, darker toward tip of femora and tibiae, base of hind femur darker. Fore tibia with distal ventral oval depression with abundant and irregularly distributed trichia; first tarsomere 1.5 times tibia length. Mid tibia with short dark trichia irregularly arranged and an apical ventral brown comb of setae, mid tarsus with dark, short, erect setae along entire length; hind tibia with trichia as in mid tibia, apex with row of dark setae. Tibial spurs 1:2:2, light brown, spurs more than three times apical width of tibiae. Tarsal claws with a large apical tooth and a smaller basal tooth. *Wing* (Fig. 4D). Length, 5.5–6.5 mm, width, 2.0 mm. Membrane hyaline with a pair of evident maculae, one more basally from anterior margin to the level of sc-r to base of medial fork, another one at distal third of wing; membrane densely covered with decumbent macrotrichia on all cells, microtrichia present; wing veins dark brown, anterior veins more strongly sclerotized. Sc complete, setose, reaching C slightly beyond level of R_4_; sc-r present, bare, slightly more basal than mid of cell r1; first sector of Rs slightly oblique, R_1_ long, reaching C at about apical fifth of wing; C extending to slightly beyond tip of R_5_; R_4_ present, cell rectangular, elongate, length almost 4 times width; R_5_ slightly curved close to apex; r-m oblique, setose. Medial and cubital veins complete basally, reaching wing margin, though slightly less sclerotized close to apex. M_1+2_ stem almost twice r-m length, M_1_ slightly divergent from M_2_ close to apex. Origin of M_4_ more basal than level of medial fork, CuA curved towards posterior margin on apical third; pseudovein sclerotized to about mid of CuA; CuP sclerotized to less than half of CuA extension. *Abdomen* (Fig. 2D). Segments 1–7 ochre-yellowish on anterior third, brown on distal two thirds, segment 8 brownish; segments cylindrical, with dark setae covering tergites and sternites. *Terminalia* (Fig. 8A–D). Dark ochre-yellowish, slightly longer than wide. Gonocoxites close to each other mesally at ventral face, with a deep incision between them; each gonocoxite ventrally with one a wide short lobe on distal margin, inner surface with abundant scattered spines and set combs of setulae; dorso-lateral projection of gonocoxite slightly rounded, on the inner surface with several short dark spines and sets of combs of setulae, also at the distal margin a long strong dark spine. Gonostylus short, not conspicuous, close to apex of ventral lobe, rounded, inner surface with scattered dark short spines. Gonocoxal bridge strongly displaced towards base of terminalia. Paramere with two distal lobes, one wide apically with scattered spines and one slender, with long dark setae. Aedeagus elongate, weakly sclerotized. Cerci digitiform, densely covered with thin setae, extending well beyond lateral tip of gonocoxite.

***Female*.** (Fig. 2E). As a male, except for the following features. Body length, 7.5–8.0 mm. *Wing* (Fig. 4E). Length, 6.5–7 mm, width, 2.3–2.5 mm. Membrane maculae at same position but darker than in males. *Terminalia* (Fig. 9A–D). Short, ochre-yellow with brown apex of sternite 8. Sternite 8 as long as wide, gonapophyses well separated from each other medially at distal margin, each gonaphysis setose, with a long, stronger seta at distal margin close to tip, long setae at distal margin medially and three thin, long setae in between. Tergite 8 covered with small setae on distal three fourth, a row of stronger, longer setae along distal margin. Vaginal furca (sternite 9) long, slender, extending anteriorly beyond anterior margin of sternite 8, rounded at anterior end. Tergite 9+10 much wider than long, covered with setulae, but no longer setae. First segment of cercus about as long as, but wider than, second segment of cercus, both setose.

#### Etymology.

The species epithet of *E. merizaldei* is dedicated to biologist Gabriel Merizalde, friend and nature lover, who for many years made possible our sampling in the wonderful forest of the private property called Nubarrones, in the Loma del Escobero neighborhood, to the south of the city of Medellín.

#### Remarks.

This species is very similar to *E. maculata*, e.g., in the color patterns on the body, likewise the setose anepisternum, and the wing membrane dark markings, although they differ in other features. The information on date of collection of the specimens of the type series suggest that this species may have its phenology related to the end of periods of great rainfall, from February to March and from June to early July. This species was found in an Andean forest dominated mainly by *Clusia multiflora* Kunth, *Magolia spinalii* (Lozano) Govaerts (Magnoliaceae), and *Ceroxyilum vogelianum* (Engel) H.Wendl. (Arecaceae).

## Discussion

These four Colombian species can be easily separated from each other, as indicated along the remarks for each species above, based on different aspects of the morphology of the species, as the color of the head and the scutum, setation of the anepisternum, wing membrane maculation and details of the wing venation, and the male terminalia. *E. maculata* and *E. merizaldei* both share several particular characters such as the body color pattern, dark vertex and palpomere, as well as the antero-dorsal short hairs of the anepisternum (Fig. 3C, D, G, H). They also share conspicuous wing membrane maculation, although with different patterns. In *E. maculata*, Sc ends at the level of the tip of R_4_ and sc-r is almost in line with the origin of Rs (Fig. 4C), while in *E. merizaldei* Sc is longer, ending in C clearly more distally in the wing (Fig. 4A, B, D), and sc-r separated from the base of cell R_1_. *E. chingaza* and *E. silvaandina* have a lighter color, more yellowish, including the head and the palpomeres (Fig. 3E, F). In both these species, the anepisternum is bare (Fig. 3I, J) and the wing venation with the Sc far from R_4_ and sc-r aligned with Rs (Fig. 4A, B). Both species were collected in the same trap, along the same period, and in the same ecosystem, but they can be discriminated from each other and from the other Colombian species by several features of the terminalia. *E. silvaandina* shows a faint darker area of the membrane along anterior margin, but does not have conspicuous maculae and M_1_, M_2_ and M_4_ are slightly curved basally close to the wing margin (Fig. 4A).

Not much can be said about the relationships between the Colombian species and other species of *Eudicrana* at this stage. Freeman (1951) proposed a division of the genus into two groups. One would gather species without microtrichia on the wing membrane and with the gonostyli articulating distally at the syngonocoxite; the other group would have microtrichia on the wing membrane, a dorso-lateral projection of the gonocoxite beyond the base of the gonostyli, and a sclerotized gonocoxal apodeme inside the male terminalia.

*E. nigriceps*, *E. obumbrata*, *E. basinerva* and *E. araucariae* would fit into the first of these two groups, while *E. claripennis*, *E. vittata*, *E. similis*, and *E. pallida* fit in the second (Freeman 1951, Matile 1991). It is interesting to mention that all these four latter species do not have maculation on the wing membrane, while all species of the first group have patterned wing membrane, but this feature was not mentioned either by Freeman (1951) or by Matile (1991) to separate these two groups.

All the four species from Colombia described here, *E. splendens* and the species from Trinidad illustrated by Lane (1960) and identified as “*E. vittata*”, have the long cerci and a lot of spines arranged in combs in the terminalia, with a short dorso-lateral projection of the gonocoxite bearing a spine distally. The dorso-lateral projection of the gonocoxite and any comb of spines are clearly missing in *E. araucariae* and apparently also missing in *E. basinerva*. This would fit the four Colombian species in the group with *E. claripennis* and *E. vittata* from Chile, the two species of Peru and *E. splendens*. This would also show that the absence of a wing maculation pattern in *E. chingaza*, in *E. vittata* and in some other species may be a secondary condition, meaning that the presence of the wing pattern does not define the species group.

*Eudicrana* specimens are rare in collections and not abundant in the field. This also applies to other Sciophilinae genera with southern temperate distribution found in Colombia (Henao-Sepúlveda et al. 2019). The species of *Eudicrana* described in this paper were collected in the Colombian high mountain ecosystems dominated by the Myrtaceae genus *Tibuchina* sp. and the oak species *Quercus humboldtii* Bompl., a genus of the family Fagaceae basically with Holarctic distribution. This reinforces the idea that this environment is a biogeographical node (in the sense of Croizat 1964), with overlap of southern and northern hemisphere elements (Oliveira and Amorim 2011, Kurina and Oliveira 2015). The specimens were collected basically at the climatic transition between dry periods and the beginning of the rainy periods, basically from November to December, from March to April and June to July.

These fragile ecosystems have been seriously damaged (Herzog et al. 2011). The description of the four rare new species occurring in these areas clearly indicates the extension of biodiversity threat in place for the high Andean ecosystems in Colombia.

## Supplementary Material

XML Treatment for Eudicrana

XML Treatment for Eudicrana silvaandina

XML Treatment for Eudicrana chingaza

XML Treatment for Eudicrana maculata

XML Treatment for Eudicrana merizaldei
